# Editorial: The role of entropy and information in evolution

**DOI:** 10.3389/fgene.2023.1269792

**Published:** 2023-09-21

**Authors:** Samuel A. Cushman

**Affiliations:** Wildlife Conservation Research Unit, Department of Biology, University of Oxford, Oxford, United Kingdom

**Keywords:** entropy, evolution, information, thermodynamics, population genetics, complexity

## 1 Introduction

The natural growth rate of populations enables them to produce more offspring that can be supported by the environment. This idea provoked Malthus (1798) to propose that there would be an inevitable crisis for human civilization given that population increases exponentially but humanity’s ability to produce food likely would only increase linearly. Darwin (1859) realized that the Malthusian concept applied to all organisms and reframed it to what he called the struggle for existence, in which individuals that are less capable of competing for limited resources would perish while those that are more fit would survive and reproduce, spreading their advantageous characters through the process he called natural selection. One Research Topic that has been largely ignored in evolutionary biology is the overarching context of thermodynamics in controlling all biological processes and the evolution of life (Cushman, 2023). In thermodynamics, a dissipative structure is a structure that emerges and is maintained by consuming free energy and efficiently increasing entropy. The second law of thermodynamics posits that all actions increase the disorder of the system in which they reside and consume useable energy in doing so. This concept has been the focus of a long history of research in the physical sciences, and entropy is a foundation of physics and chemistry (e.g., Craig, 1992; Atkins, 1994; Uffink, 2022). In the biological sciences, however, thermodynamics and entropy have been not well explored or integrated into theory and applications (e.g., Cushman, 2015; Cushman, 2021a; Cushman, 2021b; Cushman, 2021c; Cushman, 2023).

In ecology, as Cushman (2023) described, thermodynamics and entropy have been largely ignored, except for the field of macroecology (Brown, 1995) and the use of information theory, particularly in the Maximum Entropy Theory of Ecology (Harte and Newman, 2014). These lines of research are incredibly important in attempting to apply frameworks, which are related either conceptually or fundamentally, to thermodynamic entropy, to enable prediction of system properties at a macroscale based on the complex behavior of the constituent units. In both a key idea is that system properties emerge in predictable and often simple patterns from the complex interaction of myriad components (such as organisms, and the physiological processes within organisms). Despite their broad appeal and enormous impact, these lines of research have not broadly revolutionized ecological science. Cushman (2021c), Cushman (2023) proposed that this is in part because they adopt generally broad conceptual models of thermodynamic processes that do not fundamentally link physical processes to biological patterns.

Fundamentally, organisms are self-replicating dissipative structures (Cushman, 2023). To more clearly understand life, therefore, requires a thermodynamic perspective that understands organisms as the structures that emerge from the dissipation of free energy in complex systems, which have heritability and metabolism, and which therefore evolve through natural selection to more effectively reproduce their structure and more efficiently utilize and degrade free energy. Evolution, therefore, must be reconceptualized as the study of the emergence, change, propagation and adaptation of networks of self-replicating dissipative structures. However, as described in Cushman (2021c), Cushman (2023), evolutionary biology has not adopted thermodynamic perspectives or the use of entropy and the second law as an organizing framework.

The central process in all nature (including biological organisms and ecological systems) is dissipation of free energy according to the second law of thermodynamics, and evolution therefore is better conceptualized as the emergence of self-replicating dissipative structures that through natural selection become increasingly more efficient at degrading free energy and reproducing themselves. Cushman (2023) wrote that by focusing on the fundamental entity (energy), and the fundamental process (dissipation and disordering of energy and increasing of entropy), we are able to have a much clearer and powerful understanding of what life is, from the level of biochemistry, to evolution, to the nature of the organism itself, and to the emergent structures of ecosystems, food webs and communities. As Cushman (2023) noted, biology generally, and evolutionary biology in particular, came from a tradition of descriptive natural history, in which the naming and organization of organisms was of primary focus. It was natural then for Malthus and Darwin to focus on the struggle among individual organisms for survival as the focal paradigm of biology and evolution. However, a thermodynamic perspective inverts this.

Thermodynamically, organisms do not struggle in a competition for survival. Organisms emerge from the cooling of the Universe as a result of the process of dissipation of free energy. As Cushman (2023) noted, focusing on the ephemeral products of dissipation, while ignoring its fundamental unity and underlying drivers, is akin to looking at the ocean, seeing the waves, but never imagining the existence of water. A reversion of the perspective, eschewing focus on individuals, populations and species, to emphasize the flow and transmutation of energy, reveals that life is an emergent system of dissipative structures that progressively are adapted by the physical environment and their interactions to improve their ability to obtain, utilize and degrade free energy.

The scope of this Research Topic includes entropy in biological systems, information theory in evolution, linkage between complexity and entropy in biological systems, and entropy and dissipation in adaptation. The goal of the Research Topic is to focus attention on the fundamental role of thermodynamics, and in particular the second law, in biological evolution. Despite what we consider to be the central importance of this Research Topic to the future of evolutionary biology, our Research Topic was only able to successfully obtain four published contributions. This, we believe, reflects the entrenched subdisciplinary focus of evolutionary biology, with major branches focusing on molecular mechanisms of evolution, population biology, and linking genomic and epigenomic variation to fitness in different environmental contexts. All of these are highly relevant, but a paradigm shift to refocusing evolutionary biology within a thermodynamic context has not yet occurred. The contributions in this Research Topic, however, we hope will spur new attention on this subject and provoke research at the synergistic margins between the fields of molecular biology, informatics, ecology, and thermodynamics.

## 2 Contributions

There were four papers published within this Research Topic. Fibla et al. reported on geohistorical boundaries among human populations, with a particular focus on populations in the rural Catalan Pyrenees. The authors analyzed 726,718 autosomal single nucleotide variants in 435 individuals from the Catalan Pyrenees. Their results show that at a macro-geographic scale there is a consistent genetic gradient across Spain. However, their analysis also identified finer-scale, nested substructure among the sampled individuals which did not correlate with geographic barriers, such mountain ranges. The fine scale structure was correlated, conversely, with historical administrative units, which suggests that socio-cultural factors have strongly shaped the genetic diversity observed in rural populations. This result is important in investigating and separating geographical, historical and sociological factors that influence the genetic structure of human populations. This kind of spatial analysis of genetic variation is important in understanding the history of population migration and the factors that drive it, which is a fundamental question in landscape genetics, and a key to understanding the interplay between drift, gene flow and selection in evolution (Balkenhol et al., 2015). The direct linkage with thermodynamics, entropy and spatial information theory, however, is not explicit.

Johnson et al. studied the speciation of pelagic zooplankton in relation to invisible boundaries that drive isolation and enable divergent evolution in local adaptive complexes of oceanic conditions. They noted that due to the lack of obvious boundaries, speciation and population subdivision in the pelagic environment remain largely unexplained. Focusing on comb jellies (Phylum Ctenophora), the authors examined the global population structure, finding strong genetic homogeneity in some species across large regions of the ocean that appear to be subdivided by physical barriers, while also finding substructure in other species across areas showing little apparent physical discontinuity. Consistent with the findings of Lopez-Marquez et al. (2019), Lopez-Marquez et al. (2021), the authors concluded that the results suggest that oceanic currents, sea-level, and geological changes over time can act as either barriers or aids to dispersal in the pelagic environment, and that these effects are divergent among species due to historical and life history factors. They further described the emergence of a distinct lineage in the open ocean, with secondary contact zones with neighboring lineages, which is rare in apparently spatially homogeneous and geographically continuous environments, such as the open ocean. Like Fibla et al., this spatial analysis of morphological and genetic divergence in relation to the structure of the physical environment is a fundamentally important question in spatial evolutionary theory. As Cushman et al. (2014) described, understanding evolutionary processes in a heterogeneous, nonequilibrium and temporally dynamic physical environment is the fundamental challenge to taking ideal, equilibrium theories of population genetics and evolutionary biology to effective application in the real world to understand actual patterns of evolutionary change. Johnson et al. provided novel and insightful analysis of spatial patterns of evolutionary divergence in related taxa across ocean gradients. The linkage between these patterns and thermodynamic ideas was not formally explored in the paper, however.

The third paper in the Research Topic Cruz-Rosas and Miramontes more directly focused on the ideas of spatial information, complexity and biological evolution. The authors began by noting that the information that describe living systems reflects the complex interactions between internal organization and the functional performance of organisms. They then focused on the so-called origin of life problem, considering how prebiotic dynamics of matching and transfer of molecular shapes may emerge as a flow of information in prebiotic assemblages. This is a direct application of the paradigm of self-replicating dissipative structures as the focal Research Topic in evolutionary biology. The authors argued that propagation of resilient conformations could be the substrate for structural maintenance through a dynamical molecular scaffolding. They argued that this framework enables a theoretical integration from non-structured populations of polymers, to a theoretical framework that integrates the active role of these polymers in the emergence of adaptive dissipative structures, life and organisms. Response in systems that manage conformational information flow. The ideas proposed by Cruz-Rosas and Miramontes provide a framework for considering the emergence of order through the action of dissipative structures evolving increased information content through heritability and natural selection. As such, they are important and exciting contributions to the thermodynamic perspective of biological evolution as the emergence and adaptation of self-replicating dissipative structures.

This theme of self-organization of thermodynamic systems as a focal paradigm for evolutionary biology was further developed in De la Fuente et al., who explored self-organization and information processing at scales ranging from basic enzymatic activities to complex adaptive cellular behavior. They noted that understanding the origin of molecular structure and organization that underlies the complex dynamic architecture of cellular life is a fundamental question in biology and evolution. They proposed a system of biomolecular order and complexity spanning from the most elementary levels of molecular activity to the emergence of cellular systemic behaviors that integrates complexity, information theory and thermodynamic processes underlying the evolution of life. They formally focused on dissipative self-organization, which they correctly and profoundly identified as the principal source of molecular order in the cell. They then proposed that molecular information processing is the second fundamental source of biochemical order, which emerges from the biochemical dissipative self-organization. The authors suggested that effective connectivity based on Transfer Entropy is a useful framework to quantify biomolecular information flows in dissipative metabolic networks, which they proposed as a mechanism providing self-regulatory control of metabolism. They concluded that the functional structures of the cell emerge as a consequence of both main sources of order, and that quantitative analyses of dissipative metabolic networks can provide a powerful paradigm to explore the basic units of life. De la Fuente et al. also described how this dissipative metabolic structure has been verified experimentally in both prokaryotic and eukaryotic cells, and discussed using Artificial Intelligence and advanced tools of Statistic Mechanics to describe these systemic dissipative metabolic networks. This is a remarkable paper in terms of the scope and vision of its effort to integrate thermodynamic processes of dissipative structures into theoretical models of the emergence and evolution of molecular and cellular structure within the context of energetics.

## 3 Conclusion

In the thermodynamic perspective organisms are complex systems of self-replicating dissipative structures (organs, cells, organelles, biomolecules) that have emergent properties of dissipative structures themselves (Cushman, 2023). Organisms are emergent properties of a system that has evolved to most efficiently dissipate energy and increase entropy. By focusing on the fundamental entity (energy), and the fundamental process (dissipation and disordering of energy and increasing of entropy) we are able to have a much clearer and powerful understanding of what life is, from the level of biochemistry, to the nature of the organism itself, to the emergent processes of biological evolution. It has been frequently argued that the second law and thermodynamics do not apply to organisms and evolution, since evolution has produced complex structures that grow and reproduce, which seems to violate the tendency of the second law to lead to increasing disorder. This is a fundamental misunderstanding.

It is indeed the action of the second law through the process of emerging thermodynamic dissipative structures that allows all biological structure and processes. Boltzmann himself (widely seen as the father of the modern study of entropy) realized this profound implication very early in the study of thermodynamic entropy. He wrote “The general struggle for existence of animate beings is not a struggle for raw materials, these for organisms are air water and soil, all abundantly available, nor for energy which exists in plenty in the Sun and any hot body in the form of heat, but rather a struggle for entropy [exergy], which becomes available through the transition of energy from the hot Sun to the cold earth”. In this quote Boltzmann explicitly frames life as a thermodynamic system driven by entropy. He also wrote: “Thermodynamics, correctly interpreted, does not just allow Darwinian evolution; it favors it”. and “Available energy is the main object at stake in the struggle for existence and the evolution of the world”. Boltzmann emphatically stated that “In my view all salvation for philosophy may be expected to come from Darwin’s theory”. because “All structures and events correspond to the evolution of the Universe through successive states of increasing probability”. This is the deep underlying process that drives all natural change, and the emergence of all order and structure, including life itself. To rephrase the iconic last sentence of the Origin of Species into the paradigm of dissipative structures and the second law: There is a grandeur to this view of things, with its simple, undirected process underlying all creation and all structure, and whilst the cosmos goes on unwinding, according to the second law of thermodynamics, endless forms are being and will be emergent.
